# Cyclin A Degradation by Primate Cytomegalovirus Protein pUL21a Counters Its Innate Restriction of Virus Replication

**DOI:** 10.1371/journal.ppat.1003825

**Published:** 2013-12-26

**Authors:** Nicolas Caffarelli, Anthony R. Fehr, Dong Yu

**Affiliations:** Department of Molecular Microbiology, Washington University School of Medicine, Saint Louis, Missouri, United States of America; University of Alabama at Birmingham, United States of America

## Abstract

Cyclin A is critical for cellular DNA synthesis and S phase progression of the cell cycle. Human cytomegalovirus (HCMV) can reduce cyclin A levels and block cellular DNA synthesis, and cyclin A overexpression can repress HCMV replication. This interaction has only been previously observed in HCMV as murine CMV does not downregulate cyclin A, and the responsible viral factor has not been identified. We previously reported that the HCMV protein pUL21a disrupted the anaphase-promoting complex (APC), but a point mutant abrogating this activity did not phenocopy a UL21a-deficient virus, suggesting that pUL21a has an additional function. Here we identified a conserved arginine-x-leucine (RxL) cyclin-binding domain within pUL21a, which allowed pUL21a to interact with cyclin A and target it for proteasome degradation. Homologous pUL21a proteins from both chimpanzee and rhesus CMVs also contained the RxL domain and similarly degraded cyclin A, indicating that this function is conserved in primate CMVs. The RxL point mutation disabled the virus' ability to block cellular DNA synthesis and resulted in a growth defect similar to pUL21a-deficient virus. Importantly, knockdown of cyclin A rescued growth of UL21a-deficient virus. Together, these data show that during evolution, the pUL21a family proteins of primate CMVs have acquired a cyclin-binding domain that targets cyclin A for degradation, thus neutralizing its restriction on virus replication. Finally, the combined proteasome-dependent degradation of pUL21a and its cellular targets suggests that pUL21a may act as a novel suicide protein, targeting its protein cargos for destruction.

## Author Summary

Cyclins are evolutionarily conserved proteins that associate with cyclin-dependent kinases (CDKs) to regulate phosphorylation of multiple substrates to promote cell-cycle progression. Many viruses manipulate the cell cycle in order to create an environment suitable for replication; however, only few examples exist where viruses modulate cyclin activity. Here, we identified a cyclin-binding domain within the human cytomegalovirus (HCMV) protein pUL21a that confers its ability to interact with cyclin A and target it for proteasome degradation. Cyclin A promotes cellular DNA replication, which consumes important enzymes and metabolites needed for viral replication, making it important for large viruses like HCMV to block this protein's activity. In accord, the ability of pUL21a to degrade cyclin A was necessary for the virus to block cellular DNA replication and promote viral replication. Importantly, ablating cyclin A expression restored replication to a virus lacking pUL21a, demonstrating that cyclin A has the intrinsic ability to restrict viral replication, but is specifically countered by pUL21a. Together with our previous work showing that pUL21a also regulates the anaphase-promoting complex, another master cell cycle regulator, our studies have now revealed that HCMV has elegantly evolved dual functions within one protein targeting the cell cycle machinery for viral replication.

## Introduction

Human cytomegalovirus (HCMV), a widespread β-herpesvirus that establishes a lifelong infection, is capable of causing severe complications in immuno-naïve populations and immuno-compromised patients [1]. HCMV is known to use a number of mechanisms to manipulate the host cell cycle so that infected cells are arrested at the G1/S boundary. Some of these mechanisms are inhibition of Rb and activation of the E2F family of transcription factors [2]–[6], modulation of the anaphase-promoting complex (APC) [7], [8], suppression of the mini-chromosome maintenance complex (MCM) [9], and alteration of cyclin/cyclin-dependent kinase (CDK) activity [10]–[15]. It has been postulated that these regulations provide essential nutrients and cellular enzymes needed for viral DNA synthesis while preventing cellular DNA synthesis from competing for these important resources.

Cyclins are regulatory proteins that interact with CDKs to phosphorylate numerous substrates involved in cell cycle progression. It has been established that HCMV dramatically affects the levels and activity of several cyclin-CDK complexes [5], [12], [15]–[18], as well as CDK inhibitors such as p21 [13], [19], [20]. During infection, cyclins B and E are upregulated, cyclin D is largely unchanged, and cyclin A is inhibited [12], [15]–[17], whereas CDK levels are largely unaffected [15]. While the roles of cyclins B, E, and D on virus replication and virus-induced cell cycle manipulation remain to be determined, overexpression of cyclin A represses HCMV replication [14]. Cells overexpressing cyclin A had delayed IE viral gene expression, and a more noticeable block on splicing of IE transcripts as well as early and late gene expression, resulting in a multi-log reduction in viral titers. While it is likely that HCMV actively represses the expression of cyclin A to avoid its detrimental effects, the viral factor responsible and its mechanism of action remain unknown. Importantly, murine CMV (MCMV) does not actively block cyclin A expression or activity, and cyclin A overexpression does not affect MCMV gene expression [14], suggesting that the antiviral activity of cyclin A is specific to human or primate CMVs.

We have previously identified an HCMV protein, pUL21a, which is required for efficient virus growth by promoting viral DNA synthesis and late accumulation of spliced IE transcripts [21], [22]. Recently we found that this protein disrupted the APC by proteasome-dependent degradation of subunits APC4 and APC5 [7]. However, a point mutation that rendered pUL21a unable to regulate the APC (PR109-110AA) was not sufficient to alter virus growth by itself. This pUL21 point-mutant virus was attenuated only when pUL97, the second viral APC regulator, was simultaneously deleted, indicating that APC regulation is required for efficient HCMV replication. The different phenotypes between UL21a deletion virus and APC-binding point mutant virus also suggest that pUL21a must have an additional function. Here we show that pUL21a interacts with and targets cyclin A for proteasome-dependent degradation through its primate CMV conserved RxL cyclin-binding domain. This activity is completely independent of its ability to inhibit the APC. Loss of this domain results in a marked increase in cellular DNA replication and significantly attenuates virus replication. Furthermore, knockdown of cyclin A can rescue pUL21a mutant virus replication, indicating that pUL21a targets cyclin A for degradation to counter its restriction activity during HCMV replication.

## Results

### pUL21a Interacts with Cyclin A through Its Conserved RxL Domain

**Figure 1:**
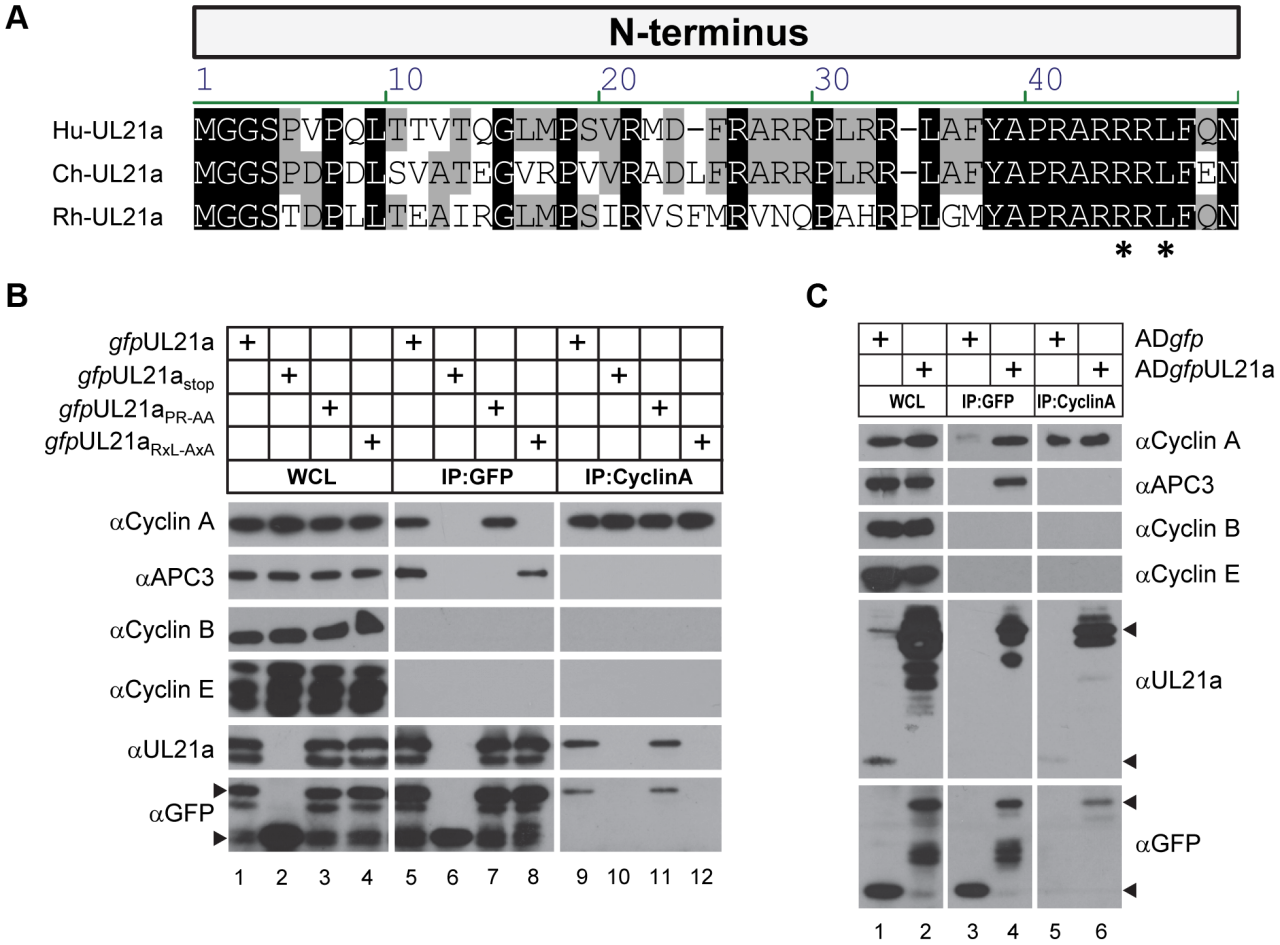
HCMV pUL21a interacts with cyclin A through its cyclin-binding domain. (A) Alignment of the N-terminus of the UL21a coding sequence from HCMV (top), ChCMV (middle) and RhCMV (bottom). Asterisks denote the residues of the RxL motif that were subject to mutagenesis. (B) 293T cells were transfected with construct expressing a GFP tagged version of wildtype (*gfp*UL21a), stop mutant (*gfp*UL21a_stop_), APC-binding domain mutant (*gfp*UL21a_PR-AA_), or cyclin-binding domain mutant (*gfp*UL21a_RxL-AxA_) pUL21a. Lysates were collected at 72 hours post infection (hpi), and immunoprecipitated with GFP or cyclin A antibody. Cell lysates and eluted proteins were analyzed by immunoblotting with indicated antibodies. Arrow indicates free GFP (bottom) or GFP-tagged pUL21a (top). Partial proteolysis was often seen with the GFP-tagged UL21a protein. (C) MRC-5 cells were infected with wildtype HCMV expressing free GFP (AD*gfp*) or GFP-tagged UL21a (AD*gfp*UL21a). Lysates were collected at 48 hpi and immunoprecipitated with GFP or cyclin A antibody. Cell lysates and eluted proteins were analyzed by immunoblotting. Arrow indicates free GFP, native pUL21a, or GFP-tagged pUL21a.

Using the Eukaryotic Linear Motif Resource (ELM) [23], we identified an arginine-x-leucine (RxL) cyclin-binding domain [24], [25] in pUL21a proteins from human CMV (HCMV), chimpanzee CMV (ChCMV), and rhesus CMV (RhCMV) (Fig. 1A). To determine whether pUL21a was able to bind to cyclins, we transfected 293T cells with constructs expressing GFP-tagged pUL21a isoforms and immunoprecipitated cell lysates with GFP antibody (Fig. 1B). Cyclin A is present in two forms: cyclin A1 that is expressed during embryogenesis and cyclin A2 that is expressed in all other dividing cells. We found that GFP-tagged pUL21a co-immunoprecipitated with cyclin A2 (termed cyclin A herein) but not cyclins B or E (lane 5). This interaction was independent of GFP as the tagged UL21a stop mutant (*gfp*UL21a_stop_) [7] failed to co-immunoprecipitate with any cyclins (lane 6). Moreover, this interaction was also independent of the binding of pUL21a to the APC, as the APC-binding mutant (*gfp*UL21a_PR-AA_) [7] co-immunoprecipitated with cyclin A even though it failed to pull down APC3 (lane 7). Importantly, mutation of the RxL domain (*gfp*UL21a_RxL-AxA_) abrogated pUL21a's ability to co-immunoprecipitate with cyclin A but not APC3 (lane 8), indicating that the RxL domain is necessary and specific for pUL21a to bind to cyclin A but not the APC. These interactions were also confirmed by reciprocal immunoprecipitation using cyclin A antibody. As expected, cyclin A co-immunoprecipitated with wildtype and PR-AA mutant pUL21a (lanes 9 and 11), but not the stop mutant or RxL mutant pUL21a (lanes 10 and 12).

To test whether pUL21a interacts with cyclin A during HCMV infection, we performed an immunoprecipitation assay on cell lysates infected with control wildtype virus expressing free GFP (AD*gfp*) or recombinant HCMV expressing GFP-tagged UL21a (AD*gfp*UL21a) (Fig. 1C). GFP tagged pUL21a co-immunoprecipitated with cyclin A and APC3 but not cyclins B and E (lane 4), and cyclin A co-immunoprecipitated with both native and GFP tagged pUL21a (lanes 5 and 6). Together, these results indicate that pUL21a uses its conserved RxL cyclin-binding domain to interact with cyclin A, and that the ability of pUL21a to bind to cyclin A or the APC is independent of each other.

### pUL21a Reduces Cyclin A Protein Levels

**Figure 2:**
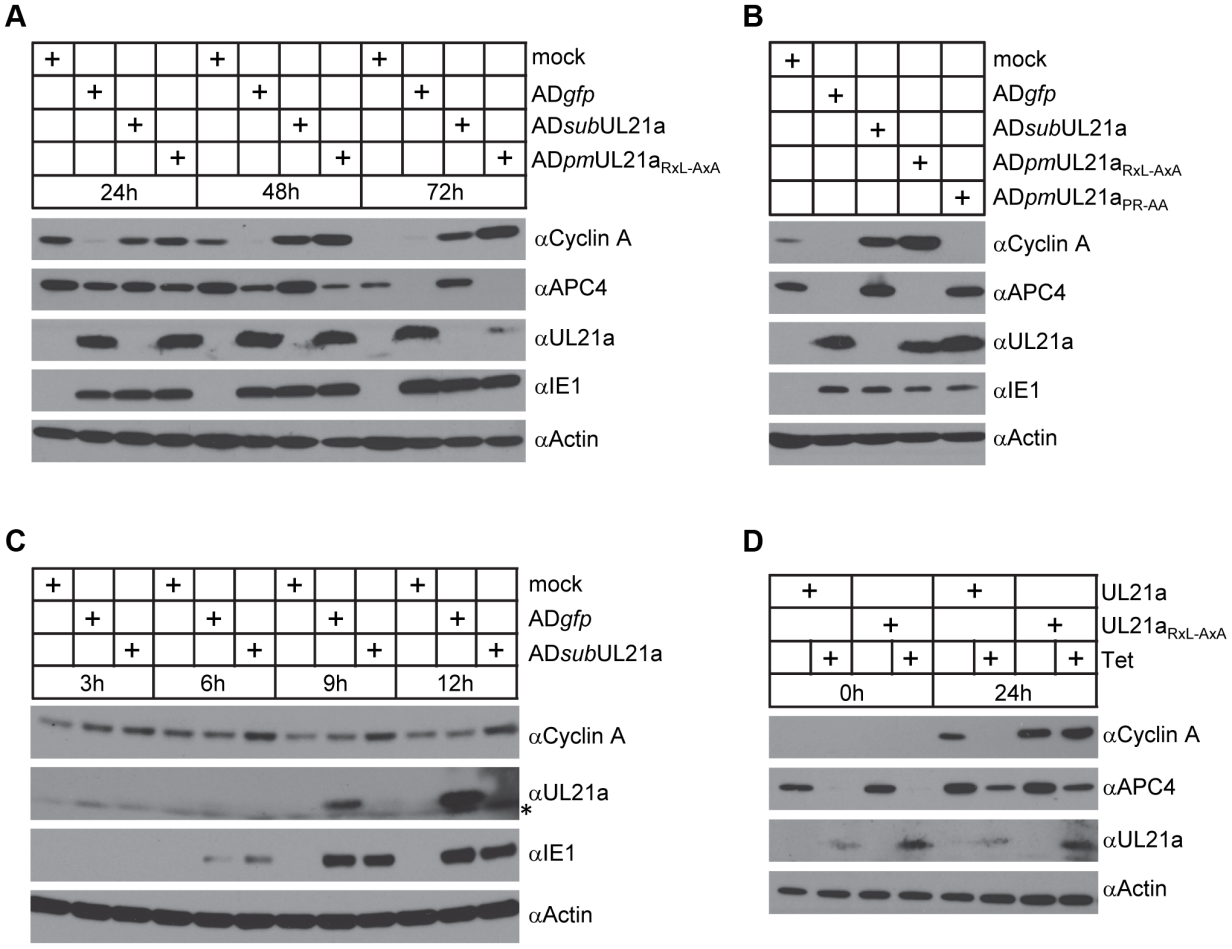
pUL21a reduces cyclin A protein levels. (A) MRC-5 cells were mock infected or infected with indicated recombinant HCMV. Cell lysates were collected at 24, 48, or 72 hpi, and analyzed by immunoblotting. IE1 and actin were used as infection and loading controls, respectively. (B) MRC-5 cells were infected as in A with indicated recombinant HCMV. Cell lysates were collected at 48 hpi and analyzed by immunoblotting. (C) MRC-5 cells were infected as in A with indicated HCMV. Cell lysates were collected at indicated times and analyzed by immunoblotting. * indicates nonspecific cross-reacting bands. (D) MRC-5 cells expressing wildtype pUL21a (UL21a) or RxL mutant (UL21a_RxL-AxA_) under a tetracycline-inducible promoter were created by lentiviral transduction. Transduced cells were serum-starved for 24 hours, then treated with or without tetracycline (1 µg/ml) for 24 hours and re-stimulated with serum to induce cyclin A expression. Cell lysates were collected at 0 or 24 hours post serum stimulation and analyzed by immunoblotting.

To test the consequence of pUL21a binding on cyclin A protein levels, we analyzed cyclin A accumulation during infection of recombinant HCMV viruses. Fibroblasts were synchronized by contact inhibition and released 24 hours before infection. This results in a sharp rise and then decrease of cyclin A levels over 72 hours. Consistent with previous studies [15], [16], wildtype virus (AD*gfp*) markedly reduced cyclin A protein levels during infection (Fig. 2A). However, cells infected with UL21a-deficient virus (AD*sub*UL21a) or cyclin binding-deficient virus (AD*pm*UL21a_RxL-AxA_) accumulated much higher levels of cyclin A. The inability of AD*pm*UL21a_RxL-AxA_ virus to reduce cyclin A accumulation was not due to an inability to induce APC subunit degradation as it degraded APC4 as efficiently as wildtype virus (Figs. 2A and 2B). This inability was also not the result of the somewhat reduced stability of mutant pUL21a at 72 hours post infection (hpi), as the mutant virus could degrade the APC efficiently at this time point. Furthermore, mutant virus AD*pm*UL21a_PR-AA_, which was unable to inhibit the APC, was still able to reduce cyclin A protein levels (Fig. 2B). Thus, not only the binding (Fig. 1), but also the ability to reduce cyclin A and APC subunit protein levels by pUL21a are genetically separable functions that do not depend on each other. Differences in cyclin A levels between wildtype and UL21a-deficient virus were apparent as early as 6–9 hours post infection (hpi) and coincides with expression kinetics of pUL21a (Fig. 2C). This suggests that the phenotype is not due to reduced levels of late gene expression seen in UL21a-deficient virus infection [22]. Together, these results indicate that pUL21a is necessary for virus-induced cyclin A reduction during HCMV infection.

**Figure 3:**
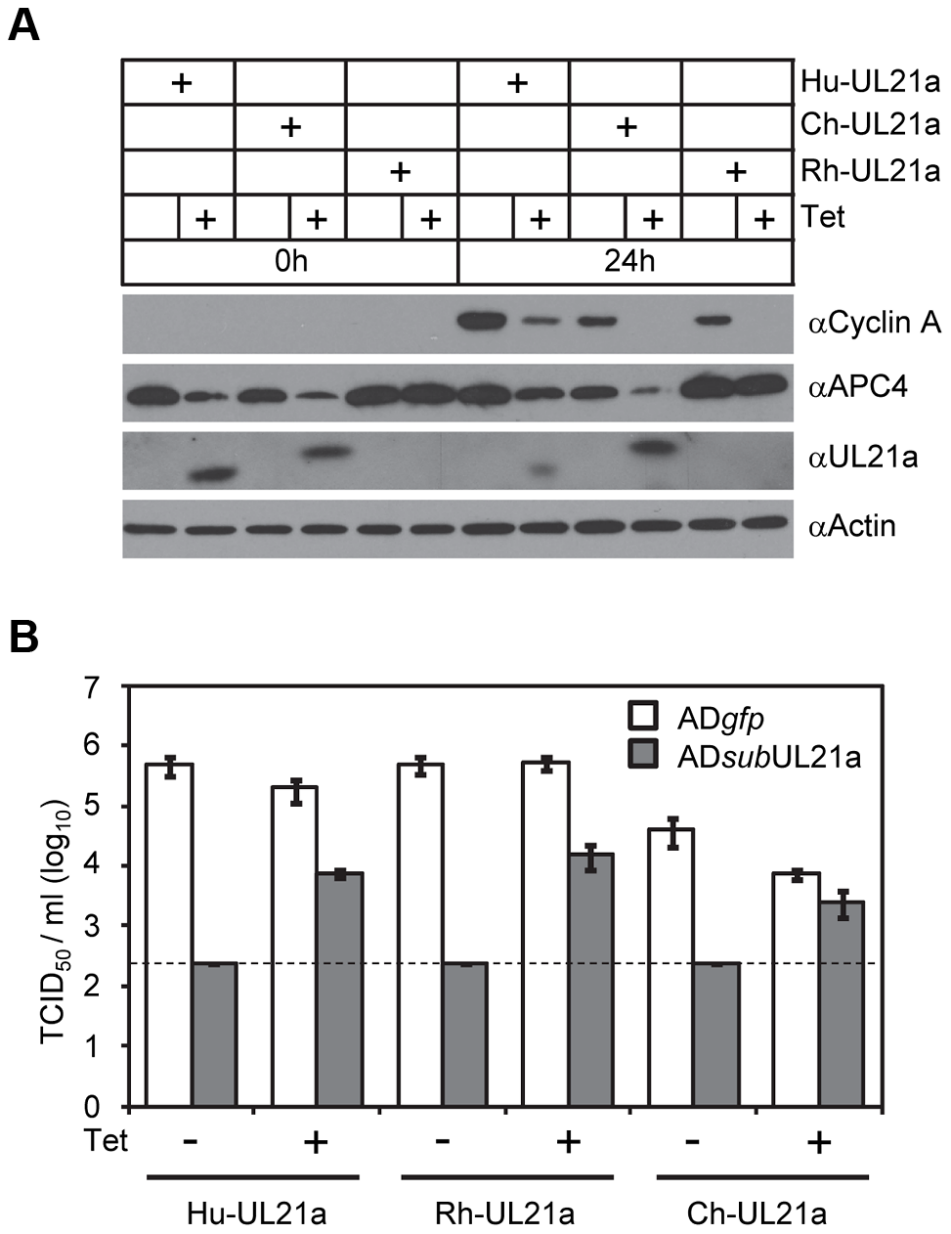
Activity to regulate Cyclin A is conserved in pUL21a of primate CMVs. (A) MRC-5 cells expressing tetracycline-inducible pUL21a from HCMV (Hu-UL21a), ChCMV (Ch-UL21a), or RhCMV (Rh-UL21a) were analyzed as described in 2D. Note that the HCMV pUL21a antibody recognized the slightly larger ChCMV pUL21a but not RhCMV pUL21a, likely due to the lack of cross-reactivity with this more divergent protein. (B) Cells expressing pUL21a variants from (A) were infected with either AD*gfp* or AD*sub*UL21a at an MOI of 0.1 TCID_50_/cell with or without tetracycline. Culture supernatant was collected 6 days post infection (dpi) and titer of cell free virus was determined by TCID_50_ assay. Dashed line indicates the limit of detection.

To test if pUL21a was sufficient to reduce cyclin A protein levels, we used a tetracycline-inducible system to over-express pUL21a in MRC-5 cells [7]. Tetracycline induced pUL21a expression, leading to the loss of cyclin A expression when compared to the non-induced control (Fig. 2D). While both wildtype and RxL mutant pUL21a reduced APC4 levels, mutant pUL21a failed to reduce cyclin A levels, further supporting the conclusion that pUL21a regulation of cyclin A but not APC4 is dependent on the RxL cyclin-binding domain. As the RxL domain is present in UL21a proteins of primate CMVs (Fig. 1A), we hypothesized that cyclin A regulation is a conserved function of pUL21a. To test this, we used the same inducible system to express UL21a proteins from ChCMV and RhCMV. Both viral proteins were able to reduce cyclin A levels (Fig. 3A). Furthermore, both proteins were able to rescue UL21a-deficient HCMV virus to levels similar to that by HCMV pUL21a (Fig. 3B). Importantly, the RhCMV UL21a protein did not degrade APC4 (Fig. 3A) but still rescued UL21a-deficient HCMV virus (Fig. 3B), providing evidence that the activity of pUL21a to inhibit cyclin A is critical for efficient HCMV replication. The ChCMV UL21a-expressing cells overall did not support HCMV infection as well as other cells did, likely due to differences in the nature of the protein and where in the cellular genome lentivirus was transduced. Together, these data strongly suggest that pUL21a-mediated reduction of cyclin A is a conserved and important function of primate CMVs.

### pUL21a Induces Proteasome-Dependent Degradation of Cyclin A

**Figure 4:**
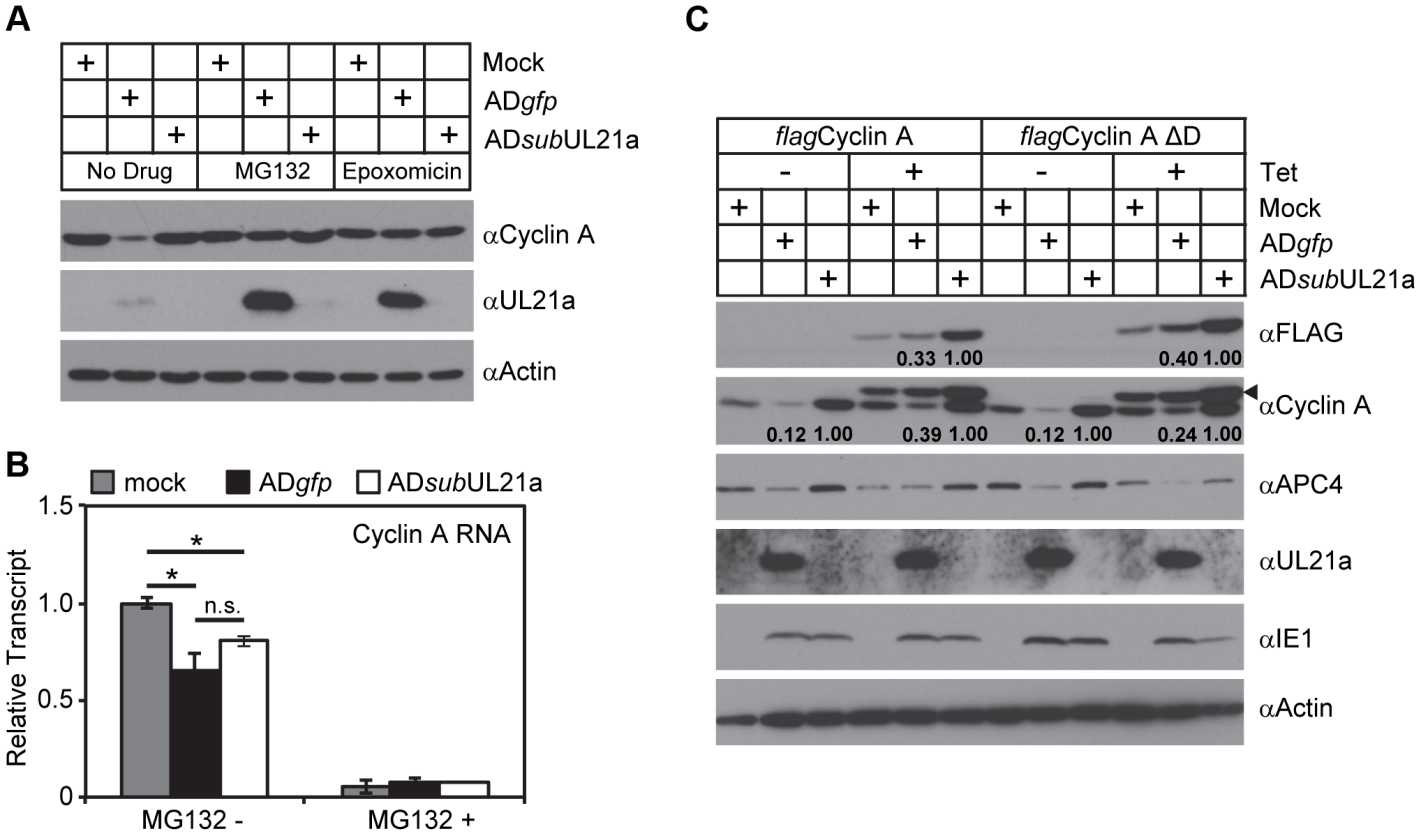
pUL21a induces proteasome-dependent degradation of cyclin A. (A) MRC-5 cells were mock infected or infected with AD*gfp* or AD*sub*UL21a. Infected cells were treated with or without MG132 or epoxomicin for 9 hours prior to collection. Cell lysates were collected at 24 hpi and analyzed by immunoblotting. (B) MRC-5 cells were infected as described in A. Cells were treated with or without MG132 at 9 hpi, and RNA was extracted at 18 hpi. Cyclin A transcripts were quantified by real-time quantitative PCR (RT-qPCR) and normalized to GAPDH. The normalized levels of cyclin A transcripts in mock infected samples were arbitrarily set to 1. *p* values were determined using the student's t test. *, *p* value <0.05; n.s. (not significant), *p* value >0.05. (C) MRC-5 cells expressing tetracycline-inducible wildtype cyclin A (*flag*Cyclin A) or D-box mutant cyclin A (*flag*Cyclin A ΔD) were created by lentiviral transduction. Cells were then infected with indicated viruses, treated with or without tetracycline, and analyzed by immunoblotting at 24 hpi. Arrow indicates FLAG-tagged cyclin A variants detected by cyclin A antibody. Protein bands of FLAG-tagged cyclin A (in anti-FLAG blot) and endogenous cyclin A (in anti-cyclin A blot) were quantitated using Image J software and normalized to AD*sub*UL21a under each condition.

We next investigated the potential mechanism of how pUL21a reduced cyclin A levels. pUL21a became detectable at 10 hpi and peaked at 24–48 hpi (Fig. 2A) [21]. Its protein levels markedly increased in the presence of proteasome inhibitors, indicating that this viral protein was inherently unstable and was targeted for proteasome degradation during HCMV infection [21]. Intriguingly, pUL21a also induced proteasome-mediated degradation of APC4 and APC5 [7]. We therefore hypothesized that pUL21a regulated cyclin A levels by inducing its proteasome-dependent degradation. To test this, we measured cyclin A protein levels in the presence of the proteasome inhibitors MG132 and epoxomicin during infection. Both MG132 and epoxomicin restored cyclin A protein levels in wildtype infection to those seen in mock and UL21a-deficient virus infected cells (Fig. 4A). While cyclin A transcript levels were reduced during infection as previously reported [12], [16], wildtype and UL21a-deficient virus had similar levels of cyclin A transcripts (Fig. 4B). Moreover, cyclin A protein levels remained high despite drastic reduction in its transcript levels following MG132 treatment (Figs. 4A and 4B). These data suggest that regulation of cyclin A by pUL21a occurs at the protein levels. Cyclin A contains a D-box motif that is necessary for recognition by the APC, the cellular E3 ubiquitin ligase that ubiquitinates and targets cyclin A for degradation during the cell cycle [14], [26], [27]. To test if the D-box motif was required for pUL21a-mediated degradation of cyclin A, we overexpressed tetracycline-inducible FLAG-tagged wildtype and D-box mutant cyclin A by lentiviral transduction in HCMV-infected cells. In the absence of tetracycline, only endogenous cyclin A was expressed, and its protein levels were reduced by almost 8-fold during wildtype virus infection as compared to those during pUL21a-deficient virus infection (Fig. 4C). In the presence of tetracycline, expressions of FLAG-tagged cyclin A was induced, and their expression was driven by the CMV promoter that was further potentiated during CMV infection. Therefore, unlike endogenous cyclin A, the levels of FLAG-tagged cyclin As were higher in HCMV-infected cells relative to those in mock-infected cells (Fig. 4C). Nonetheless, in the presence of tetracycline, pUL21a was able to similarly reduce protein levels of endogenous cyclin A, overexpressed FLAG-tagged wildtype, and D-box mutant cyclin A during HCMV infection (Fig. 4C). We noted that cyclin A reduction by pUL21a in the presence of tetracycline was less pronounced than that in the absence of tetracycline (i.e. ∼8-fold vs. ∼3-fold) (Fig. 4C). The lesser reduction of cyclin A in the presence of tetracycline was likely due to overexpression of cyclin A which overwhelmed the capacity of pUL21a to target it for degradation. Overall, the ability of pUL21a to degrade D-box cyclin A mutant, along with independent binding and degradation of the APC and cyclin A by pUL21a (Figs. 1 and 2), suggest that pUL21a-mediated degradation of cyclin A is independent of the APC.

Together, our data show that pUL21a regulates cyclin A by inducing its proteasome-dependent degradation, and that this regulation is likely independent of the APC, the cellular E3 ligase known to regulate cyclin A ubiquitination and degradation.

### The pUL21a Cyclin-Binding Domain Is Necessary to Prevent Cellular DNA Synthesis during HCMV Infection

**Figure 5:**
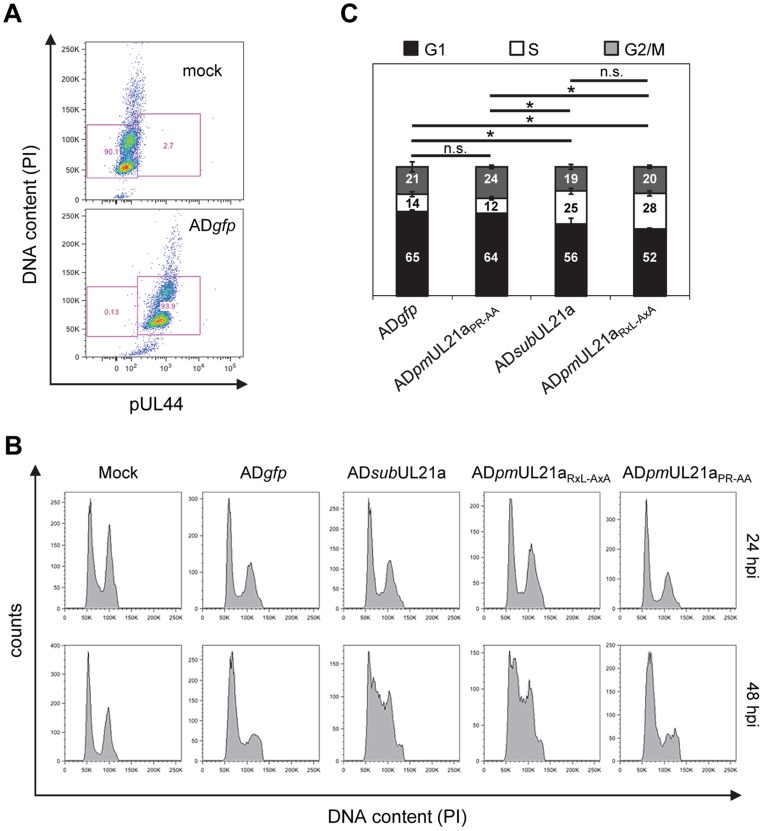
The pUL21a cyclin-binding domain is essential for HCMV to prevent cellular DNA synthesis. MRC-5 cells were infected with indicated viruses in the presence of viral DNA replication inhibitor phosphonoacetic acid (PAA). Cells were collected at 24 and 48 hpi, fixed, and co-stained with propidium iodide (PI) and antibody to viral protein pUL44. Cell cycle profiles were analyzed by flow cytometry. (A) The PI and pUL44 staining profiles of representative mock- and wildtype virus- infected cells at 48 hpi. Also shown is gating used to separate pUL44-positive (infected) and pUL44-negative (uninfected) cells. (B) Both 24 and 48 hpi DNA content profiles of mock-infected cells or pUL44-positive, virus-infected cells. (C) Percentage of pUL44-positive, virus-infected cells in G1, S, or G2/M phase at 48 hpi based on DNA content. *p* values were based on the numbers of S phase cells and determined using the student's t test. *, *p* value <0.05; n.s. (not significant), *p* value >0.05. Shown are data from two independent experiments.

HCMV inhibits cellular DNA synthesis [28], and cyclin A is a critical factor for promoting cellular DNA synthesis. We hypothesized that pUL21a regulation of cyclin A was important to prevent cellular DNA synthesis during HCMV infection. To test this, we infected MRC-5 fibroblasts with recombinant virus and measured cellular DNA synthesis by flow cytometry. Cells were gated by pUL44 expression to distinguish infected cells from uninfected cells (Fig. 5A). No differences in cellular DNA content were seen at 24 hpi (Fig. 5B). However by 48 hpi, while wildtype and APC-binding mutant viruses continued to inhibit cellular DNA synthesis, allowing only 12–14% of cells to enter S phase, UL21a-deficient and RxL mutant viruses failed to do so, resulting in nearly twice as many cells in S phase (25–28%) (Figs. 5B–C). We conclude that pUL21a-mediated cyclin A degradation is one mechanism used by HCMV to block cellular DNA synthesis during infection.

### pUL21a Counteracts the Antiviral Effects of Cyclin A

**Figure 6:**
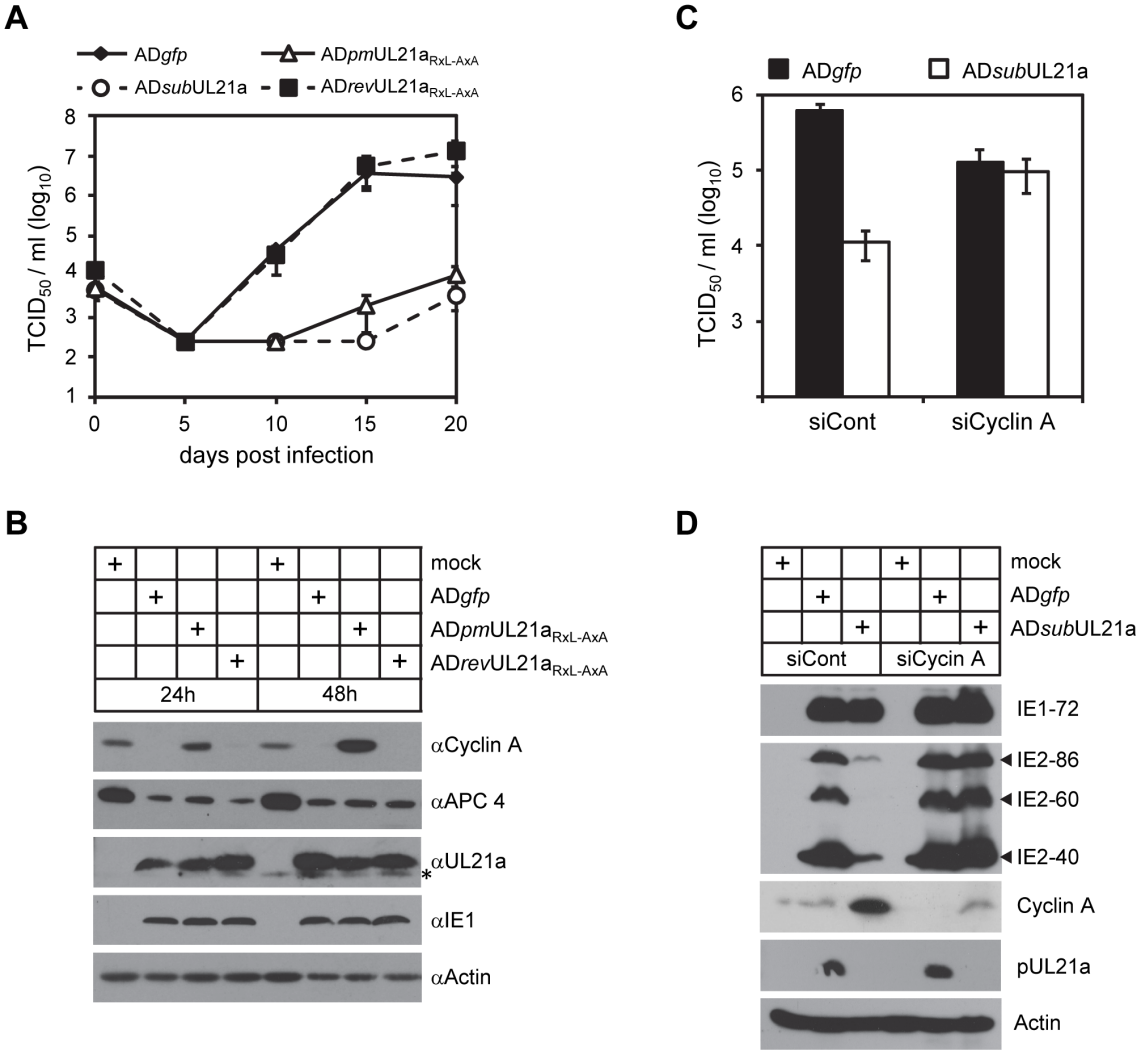
Depletion of cyclin A alleviates the requirement of pUL21a for HCMV replication. (A) MRC-5 cells were infected with indicated viruses at an MOI of 0.01 TCID_50_/cell and production of cell free virus was determined by TCID_50_ assay at indicated time points. (B) MRC-5 cells were infected with indicated viruses at an MOI of 3, and cell lysates were collected at 24 and 48 hpi and analyzed by immunoblotting. * indicates nonspecific cross-reacting bands. (C) MRC-5 cells were treated with siRNA against cyclin A (siCyclin A) or luciferase control (siCont), and then infected with indicated virus at an MOI of 3. At 96 hpi, production of cell free virus was determined by TCID_50_ assay, and (D) cell lysates were analyzed by immunoblotting.

In the final experiments, we tested the consequence of pUL21a-mediated cyclin A degradation on HCMV replication in fibroblasts. It was previously shown that cyclin A overexpression inhibited HCMV replication by several logs and was responsible for the block in lytic gene expression during the S/G2 phases [13], [14]. Consistent with this, UL21a-deficient and RxL mutant viruses were also attenuated, with a growth defect of ∼3 logs relative to wildtype and marker rescued virus (Fig. 6A). In addition, marker rescued virus reduced cyclin A protein levels as efficiently as wildtype virus, indicating that the defect in cyclin A regulation during RxL mutant virus infection is not due to second-site mutations (Fig. 6B). These data together suggest that cyclin A degradation by pUL21a is necessary for effective virus replication. To provide additional proof testing this hypothesis, we knocked down cyclin A in MRC-5 fibroblasts and infected these cells with wildtype and UL21a-deficient virus. Cyclin A knockdown rescued virus production and IE2 expression (IE2-86, IE2-60, and IE2-40) of UL21a-deficient virus to levels equivalent to wildtype virus (Figs. 6C–D). Wildtype virus showed slightly reduced growth in the presence of cyclin A knockdown, likely reflecting an off-target effect of this siRNA or a consequence of the loss of an essential host protein. Together, these data suggest that cyclin A degradation by pUL21a is necessary for effective virus replication.

We conclude that pUL21a antagonizes the cyclin A-mediated restriction on HCMV infection by binding to and promoting proteasome degradation of this prominent cell cycle regulator.

## Discussion

Here we describe a novel function for the HCMV protein pUL21a to bind to the important cell cycle regulator cyclin A and direct it for proteasome degradation, which represents a novel viral mechanism to combat this host factor. This interaction is mediated by a highly conserved RxL cyclin-binding domain that is present in pUL21a proteins of all primate CMVs tested in this study. pUL21a from ChCMV and RhCMV not only similarly reduce cyclin A levels, they also rescued the growth defect of pUL21a-deficient HCMV virus. pUL21a is a highly unstable protein subject to rapid proteasome-mediated degradation [21]. Intriguingly, it binds to two major cell cycle regulators, the anaphase-promoting complex (APC) [7] and cyclin A, and targets both for degradation in a proteasome-dependent manner. Finally, this pUL21a-mediated cyclin A degradation bears an important consequence on virus replication. Mutation in the RxL cyclin-binding domain of pUL21a destroys the ability of HCMV to inhibit cyclin A, resulting in unchecked cellular DNA synthesis and severe attenuation of virus growth. Importantly, growth of the UL21a-deficient virus can be rescued by knocking down cyclin A, providing the definitive proof that pUL21a targets cyclin A for degradation in order to antagonize its innate restriction on HCMV replication.

**Figure 7:**
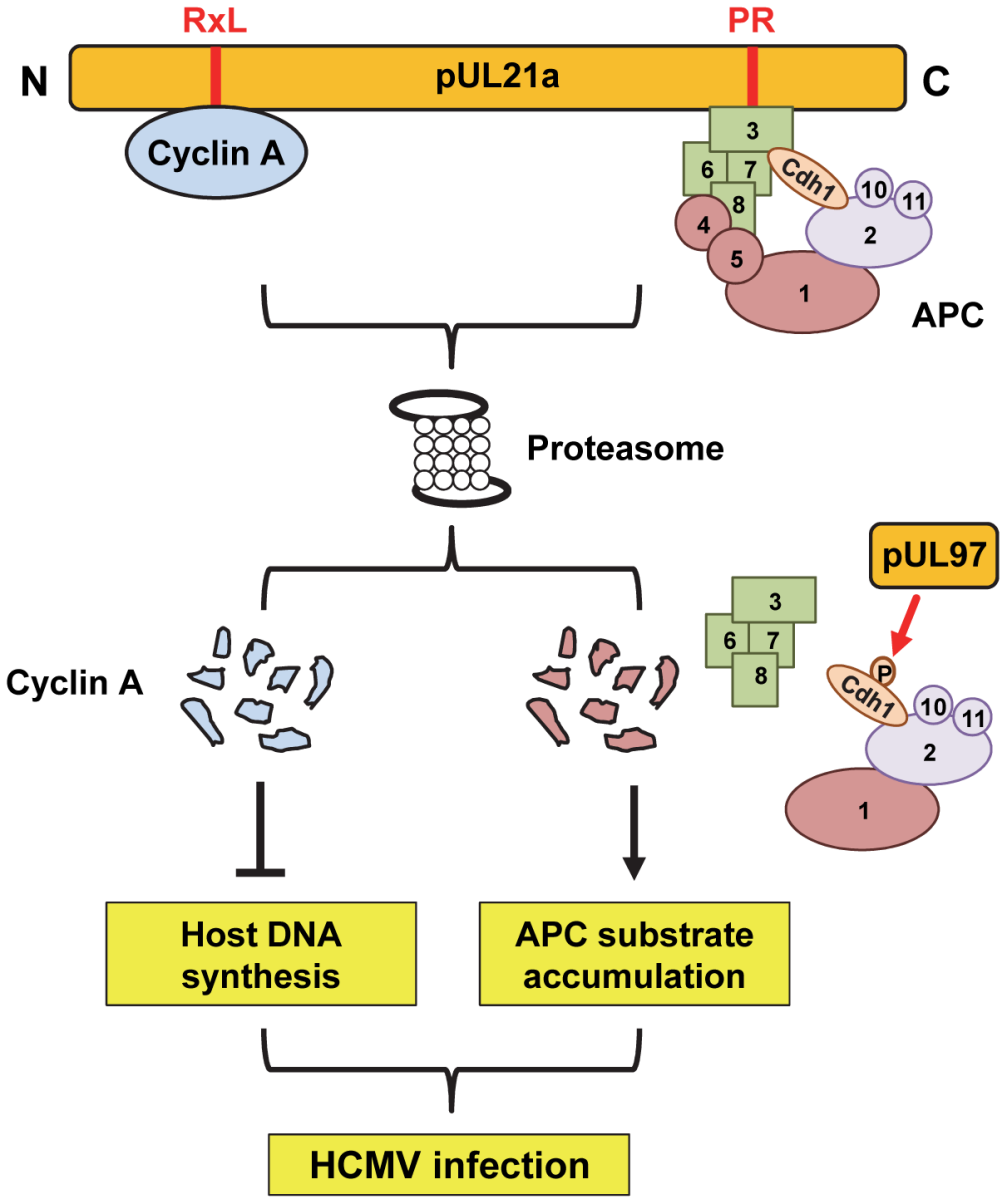
Model for dual roles of pUL21a in HCMV infection. pUL21a independently binds to cyclin A or the anaphase-promoting complex (APC) through the RxL or PR domain, respectively, and targets each for proteasome-dependent degradation. pUL21a-induced degradation of the APC bridge, in concordance with pUL97-induced phosphorylation of Cdh1, leads to an increase in APC substrates, which helps to create a favorable, S-phase like cellular environment for DNA synthesis. However, pUL21a-induced degradation of cyclin A allows HCMV to specifically prevent host DNA synthesis. Together, these two independent activities of pUL21a help to subvert host cells for efficient HCMV growth.

Our work suggests that pUL21a exploits the proteasome pathway in a way that it independently regulates APC and cyclin A protein levels. We have recently reported that pUL21a binds to and targets APC subunits 4 and 5 for degradation, leading to the dissociation of this complex [7]. Now we show that a pUL21a point mutant unable to target APC4 and APC5 (i.e. PR-AA mutation) is still able to target cyclin A for degradation, and conversely, a point mutant unable to target cyclin A (RxL-AxA) is still able to target APC4 for degradation. We conclude that these two functions of pUL21a are independent of each other and propose the model where pUL21a uses two distinct domains to uniquely interact with these cell cycle regulators (Fig. 7). Whether pUL21a uses separate or similar mechanisms to target them for proteasome degradation remains to be determined. Regardless, these two functions of pUL21a lead to distinct phenotypic effects (Fig. 7). It has been known that HCMV promotes cells into a pseudo G1/S phase by inducing expression of S-phase genes but simultaneously blocking cellular DNA synthesis. pUL21a-induced degradation of APC subunits, along with pUL97-mediated phosphorylation of the APC co-activator Cdh1 [8], leads to increased levels of APC substrates, which would promote entry into S phase [29]. However, inhibition of the APC could also increase cyclin A levels, which is detrimental to HCMV replication. Therefore, HCMV has developed a second function within pUL21a, which is to promote cyclin A degradation. This will antagonize the antiviral activity of cyclin A, inhibit cellular DNA synthesis, and phenotypically prevent infected cells from entering S phase. Thus the virus has elegantly evolved two mechanisms within one protein to harness the benefits of inhibiting the APC as well as overcoming any detrimental consequences of such regulation.

How does pUL21a target cyclin A for proteasome degradation? Cyclin A is normally targeted for ubiquitination and proteasome degradation during M phase of the cell cycle. Preliminary work suggests a modest increase in cyclin A ubiquitination in the presence of pUL21a (data not shown), so it is possible that pUL21a may have a role in cyclin A ubiquitination. If so, as pUL21a contains no domain indicating it as an E3 ligase, it could recruit an E3 ligase to ubiquitinate cyclin A or block the activity of a deubiquitinase (DUB). Alternatively, it is also entirely possible that pUL21a may directly target cyclin A to the proteasome in a ubiquitin-independent manner. Cyclin A is normally targeted by the APC for ubiquitination and proteasome degradation. However, inhibition of the APC by pUL21a and pUL97 [7], [8], along with our present data showing that pUL21a can mediate cyclin A degradation in the absence of its APC recognition motif (D-box), suggests that cyclin A degradation during HCMV infection is APC-independent. Interestingly, pUL21a itself is rapidly degraded by the proteasome in a ubiquitin-independent manner, and is highly sensitive to proteasome inhibition [21]. It is tempting to speculate that pUL21a may act as a novel “suicide” protein that delivers substrates to the proteasome and is degraded with them. Mechanistically, pUL21a could directly bind to the proteasome, or could be recognized as an unfolded protein due to its proline-rich, predicted unstructured C-terminus tail [7], and transported to the proteasome by chaperones. Additional biochemical, genetic, and structural analysis will be critical to testing these hypotheses.

What is the mechanism for cyclin A to restrict virus replication? Cyclin A and its concomitant CDK's phosphorylate a number of substrates that promote cellular DNA synthesis. It is possible that active cellular DNA synthesis would limit the resources available for viral genome amplification and interfere with the process of viral DNA replication, ultimately attenuating virus growth. Thus, pUL21a mutants may provide us with a tool to test the long held belief that cellular DNA replication is detrimental to herpesvirus replication. Alternatively, a substrate or set of substrates of cyclin A may also have more direct and specific antiviral activity that blocks HCMV replication. This pUL21a-mediated cyclin A regulation appears to be unique to CMVs of high mammals, such as primates, as murine CMV does not encode a UL21a homologous protein. It is possible that murine CMV has evolved alternative mechanisms to arrest the cell cycle, or that its shorter life cycle (∼32 hours) circumvents the need to inhibit cyclin A activity of host cells. Consistent with this, murine CMV upregulates cyclin A levels and initial viral gene expression is unaffected by cyclin A overexpression [14], highlighting an interesting and striking difference between closely related virus families.

Finally, the discovery of pUL21a as a cyclin A modulator can also provide a useful tool to delineate the activity of this important molecule in cell biology. Only recently have large-scale screenings been used to systematically identify cyclin A substrates in mammalian cells [30]. pUL21a may be used to confirm these recently identified cyclin A substrates, explore the role of these substrates in cellular DNA synthesis, or discover additional substrates that play roles in cellular DNA synthesis and proliferation. Viral systems have been instrumental in many seminal discoveries in the history of cell biology. Novel virus-encoded regulators such as pUL21a can be powerful tools to probe the biology of host cells that are otherwise difficult to study.

## Materials and Methods

### Cell Culture

Human foreskin fibroblasts (HFFs), primary embryonic lung fibroblasts (MRC-5), and 293T cells were propagated in Dulbecco's modified Eagle medium (DMEM) containing 10% fetal bovine serum and penicillin-streptomycin. Expression constructs were transfected into 293T cells with 1 mg/ml polyethyleneimine (PEI, Polysciences) in Opti-MEM media.

### Plasmids and Reagents

Primers used in this study are listed in Table 1. Point mutant UL21a sequences were constructed using PCR with the desired mutations incorporated into complementary primers. To create expression constructs, the RxL mutant UL21a PCR fragment was digested with restriction enzymes Bgl II and EcoR I, and ligated into a pLPCX (Clontech)-derived over-expression vector with an N-terminal GFP tag to create retroviral expression construct pYD-C760; or digested with Sal I and EcoR I and ligated into pYD-C639, a pLKO-based lentiviral vector under a tetracycline-inducible CMV-TetO_2_ promoter [31] (generous gift from Roger Everett, University of Glasgow), to create lentiviral expression construct pYD-C762. RhCMV UL21a was amplified from RhCMV BAC [32], ChCMV UL21a was constructed from long overlapping primers, and FLAG tagged wildtype cyclin A as well as D-box mutant cyclin A (cyclin A ΔD) were amplified from plasmids provided by Anindya Dutta (University of Virginia School of Medicine) [26]. They were all cloned into pYD-C639 to create lentiviral expression constructs. All other constructs used in this study have been previously described [7].

**Table 1:** Primers used in this study.

Purpose	Primer sequence^a^	Construct name
Overlapping PCR primers to create UL21a RxL-AxA mutation	5′cgagctcgt*gcg*agg*gct*ttccaaaatc3′ 5′gattttggaa*agc*cct*cgc*acgagctcg3′	Non applicable
Overlapping primers to create template for amplifying ChUL21a	5′atgggaggcagtcccgaccccgatctctcggtggccaccgagggtgtgaggcccgtggtccgcgcggacctgttccgagcccgccggcccctgc3′ 5′cctgaaggtgctggtgatgctgatggtagttctcaaaaagccggcgccgggcccgcggagcatagaaagccagacggcgcaggggccggcgggct3′ 5′tcaccagcaccttcaggtcccgccgccgattcatcggatcgtcgctgtgcccggaggggacgaggaagcgataccgatggacctgccgagggaga3′ 5′tgggcggaacgtcgtccaacagcagcaccagcgggttgggcagcgggcggtcgggcggtatatcggcggcgacctggatctccctcggcaggtcc3′ 5′ttaaaaatgttcccagttctcttcgcgtatcacggggtactcgcggggcactcggaaaggagcgaagccgggcggcatgggcggaacgtcgtcc3′	Non applicable
PCR primers to clone UL21a into pLKO vector	5′gtcgaccgagatgggaggtagccctgttcc3′ 5′ggaattcttaaaactggtcccaatgttctt3′	pLKO-UL21a pLKO-UL21a RxL-AxA
PCR primers to clone RhUL21a into pLKO vector	5′gtcgaccgagatgggaggcagcaccga3′ 5′ggaattcttaagcgtgtgcttcttcat3′	pLKO-RhUL21a
PCR primers to clone ChUL21a into pLKO vector	5′gggtcgacatgggaggcagtcccgac3′ 5′gggaattcttaaaaatgttcccagttctc3′	pLKO-ChUL21a
PCR primers to clone 3xFlag-Cyclin A into pLKO vector	5′gcgtcgacatggactacaaagaccatgacggtgatttaaagatcatgatatcgattacaaggatgacgatgacaagttgggcaactctgcgcc3′ 5′gcgaattcttacagatttagtgtctctggtggg3′	pLKO-3xFlag-Cyclin A pLKO-3xFlag-Cyclin A ΔD-box
PCR primers to clone HA-Ubiquitin into pLKO vector	5′gcgctagcatgtacccatacgacgtccca3′ 5′gcgaattctcacccacctcttagtctt3′	pLKO-HA-Ubiquitin
Primer pairs to quantify cyclin A transcript levels by RT-qPCR	5′gcatgtcaccgttcctcctt3′ 5′cagggcatcttcacgctctat3′	Non applicable
Primer pairs to quantify GAPDH transcript levels by RT-qPCR	5′ctgttgctgtagccaaattcgt3′ 5′acccactcctccacctttgac3′	Non applicable

^a^ Restriction sites are underlined; base pair changes to introduce mutations are underlined and in italics.

Lentivirus was produced by PEI transfection of corresponding expression constructs as described above, along with appropriate packaging plasmids, into 293T cells. To create stable expression cells, MRC-5 cells expressing GFP-TetR [7] were transduced with lentivirus, and selected with 2 µg/µL puromycin (Sigma-Aldrich) to produce stable cells expressing various forms of UL21a, FLAG-cyclin, or HA-ubiquitin under the CMV-TetO_2_ promoter.

To knock down cyclin A by RNAi, MRC-5 cells were transfected with siGENOME siRNA against cyclin A or luciferase control (Thermo Scientific), using the procedure of siLENTFECT (Bio-Rad) according to manufacturer's instructions. Cells were left in serum-free medium and infected 48 hours later in serum-containing medium.

Primary antibodies used included anti-β actin (AC-15, Abcam); anti-GFP (3E6 and A6455, Invitrogen); anti-APC3 (35/CDC27, BD Biosciences); anti-APC4 (A301-176A, Bethyl laboratories); anti-UL21a [21]; anti-IE2 (mAB8140, Chemicon); anti-UL44 (10D8, virusys); anti-Cyclin A (B-8 and H-432, Santa Cruz); anti-cyclin B (GNS1, Thermo Scientific); anti-cyclin E (HE12, BD Biosciences); anti-FLAG (F1804, Sigma); anti-HA (16B12, Covance); and anti-IE1 (generous gift from Thomas Shenk, Princeton University).

### Recombinant HCMV Virus

BAC-HCMV clones used in the present study were constructed using PCR-based two-step linear recombination as previously reported [33]. pAD*gfp*, which carried the genome of HCMVAD169 strain and a simian virus 40 early promoter-driven GFP gene in place of the viral US4–US6 region, was used to produce wildtype virus AD*gfp* [34]. pAD*pm*UL21a_RxL-AxA_, which carried the point mutation RxL42-44AxA in the UL21a coding sequence, and pAD*rev*UL21a_RxL-AxA_, in which RxL42-44AxA mutation was subsequently repaired, were used to produce UL21a cyclin-binding domain point mutant virus AD*pm*UL21a_RxL-AxA_ and its marker rescued virus AD*rev*UL21a_RxL-AxA_, respectively. These recombinant BAC clones were confirmed by PCR, restriction digest, and sequencing. All other recombinant BAC-HCMV clones used were described previously [7]. Recombinant HCMV AD169 viruses were reconstituted from transfection of corresponding BAC-HCMV clones as previously described [33]. Viral stocks were harvested from infected cell culture supernatant and concentrated by ultracentrifugation through 20% D-sorbitol. Virus titers were determined in duplicate by tissue culture infectious dose 50 (TCID_50_) assay on HFFs. Relative viral genome numbers were determined by extracting virion DNA and performing real-time quantitative PCR (qPCR) with either a taqman probe and primers specific to viral gene UL54, or with SYBR green and primers to UL32 [21].

### HCMV Infection

Confluent MRC-5 cells were split, and 24 hours later infected with HCMV at an input genome number equivalent to that of 3 infectious units of wildtype virus/cell, unless otherwise indicated. Cells were inoculated for 1 hour with virus and then replenished with fresh media. For cell cycle profiling, cells were treated with phosphonoacetic acid (PAA, 100 µg/ml, Sigma-Aldrich) immediately after infection. For proteosome inhibition experiments, cells were treated with MG132 (20 µM, Santa Cruz) or epoxomicin (40 µM, Santa Cruz). Virus titers in the supernatant of infected cultures were determined by TCID_50_ assay.

### Protein Analysis

For immunoprecipitation, 293T and MRC-5 cells were lysed in NP40 buffer (0.5% NP40, 50 mM Tris-Cl pH 8.0, 125 mM NaCl) and EBC2 buffer (0.5% NP40, 50 mM Tris-Cl pH 8.0, 300 mM NaCl), respectively. Lysis buffers were supplemented with protease and phosphatase inhibitors (Roche and Sigma-Aldrich, respectively). Mouse anti-GFP antibody (3E6, Invitrogen) or mouse anti-Cyclin A antibody (B-8, Santa Cruz) was conjugated to protein A dynabeads (Invitrogen) with BS^3^ (Thermo Scientific) according to manufacturer's instructions. Cleared cell lysates were incubated with conjugated dynabeads by gentle rotation at 4°C. Beads were washed once with lysis buffer and twice with PBS, and bound proteins were eluted in reducing sample buffer (200 mM Tris pH 6.8, 6% SDS, 12% β-mercaptoethanol, 18% glycerol) by incubating at 55°C. For whole cell lysate control (WCL), cell lysates were similarly mixed with reducing sample buffer and incubated at 90°C for 5 minutes.

For immunoblotting, cells were lysed in reducing sample buffer containing protease and phosphatase inhibitors. Proteins from equivalent cell numbers were resolved on a SDS poly-acrylamide gel, transferred to a polyvinylidene difluoride membrane, hybridized with primary antibody, reacted with horseradish peroxidase-conjugated secondary antibody, and visualized using chemiluminescent substrate (Thermo Scientific).

### Analysis of Cellular DNA Content

Cells were trypsinized, washed with PBS, and fixed in 70% ethanol. Fixed cells were double stained with propidium iodide (PI) for DNA and with anti-pUL44 antibody to identify infected cells, and analyzed by flow cytometry. Cells were gated for pUL44 and PI staining, and percentages of cells in each cell cycle compartment were calculated using FlowJo software.

### Reverse Transcription Coupled-Quantitative PCR Analysis (RT-qPCR)

Total RNA was extracted with TRIzol (Invitrogen), treated with TURBO DNA-free (Ambion) to remove DNA contaminants, and reverse transcribed with random hexamer primers using the High Capacity cDNA RT Kit (Applied Biosystems). The cDNA was quantified by qPCR using SYBR green SYBR Advantage qPCR Premix (Clontech) with primers for the cellular genes Cyclin A or GAPDH (glyceraldehyde-3-phosphate dehydrogenase) (Table 1). cDNA from six arbitrary samples were mixed together and serially diluted to generate a standard curve used to calculate the relative amount of specific RNA.
